# Bone turnover markers are associated with bone density, but not with fracture in end stage kidney disease: a cross-sectional study

**DOI:** 10.1186/s12882-017-0692-5

**Published:** 2017-09-06

**Authors:** Hanne Skou Jørgensen, Simon Winther, Morten Bøttcher, Ellen-Margrethe Hauge, Lars Rejnmark, My Svensson, Per Ivarsen

**Affiliations:** 1 Department of Renal Medicine, Aarhus University Hospital, Palle Juul-Jensens Boulevard 99, 8200 Aarhus N, Denmark; 2 Institute of Clinical Medicine, Aarhus University, Aarhus, Denmark; 3 Department of Cardiology, Aarhus University Hospital, Aarhus, Denmark; 4 Department of Internal Medicine, Hospital Unit West, Herning, Denmark; 5 Department of Rheumatology, Aarhus University Hospital, Aarhus, Denmark; 6 Department of Endocrinology and Internal Medicine, Aarhus University Hospital, Aarhus, Denmark; 7 Department of Nephrology, Division of Medicine, Akershus University Hospital, Oslo, Norway

**Keywords:** Bone density, Bone remodeling, Chronic kidney disease, Fracture, Osteoporosis, Renal osteodystrophy

## Abstract

**Background:**

Fracture risk is increased in chronic kidney disease (CKD), but assessment of bone fragility remains controversial in these patients. This study investigated the associations between bone turnover markers, bone mineral density (BMD), and prevalent fragility fracture in a cohort of kidney transplantation candidates.

**Methods:**

Volumetric BMD of spine and hip was measured by quantitative computed tomography. Parathyroid hormone (PTH), bone-specific alkaline phosphatase, procollagen type-1 N-terminal propeptide, tartrate resistant alkaline phosphatase, and C- and N-terminal telopeptides of type 1 collagen were analyzed from fasting morning blood samples. Fragility fractures included prevalent vertebral fractures and previous low-trauma clinical fractures.

**Results:**

The fracture prevalence was 18% in 157 adult kidney transplant candidates. Fractured patients had reduced BMD and *Z*-score at both spine and hip. Levels of bone turnover markers were significantly higher in patients on maintenance dialysis than in pre-dialysis patients; but did not differ between patients with and without fracture. There were strong, positive correlations between PTH and all bone turnover markers. PTH was negatively associated with *Z*-score at lumbar spine and total hip; in contrast, bone turnover markers were only negatively associated with total hip *Z*-score.

**Conclusions:**

Bone turnover markers were negatively associated with bone density, but not associated with prevalent fracture in kidney transplantation candidates. The role of bone turnover markers in assessing bone fragility in CKD will require further investigation.

**Trial registration:**

This study was registered at ClinicalTrials.gov with identifier NCT01344434.

**Electronic supplementary material:**

The online version of this article (10.1186/s12882-017-0692-5) contains supplementary material, which is available to authorized users.

## Background

Fracture risk is increased in chronic kidney disease (CKD) [1, 2], and remains high after kidney transplantation [3]. In the general population, bone mineral density (BMD) is used to assess fracture risk [4, 5], but this approach is not recommended in late stages of CKD [6]. In addition to a loss of bone quantity, which can be measured by BMD, patients with CKD suffer changes in bone remodeling which may be inappropriately high or low, and with or without mineralization defects [7]. A transiliac bone biopsy remains the gold standard for evaluating changes in bone remodeling, but biochemical markers of bone turnover have been proposed as non-invasive alternatives [8].

Bone turnover markers are proteins produced by active bone cells or fragments of collagen released into the circulation during bone remodeling. High levels may predict fracture in men and women with normal kidney function [9], and several markers are recommended for use in monitoring the response to anti-resorptive therapy [10]. A previous study found strong associations between the levels of bone turnover markers and prevalent fracture in patients with pre-dialysis CKD [11]. In a longitudinal study, increasing levels of bone turnover markers predicted loss of BMD over time [12].

The clinical usefulness of bone turnover markers in CKD remains unclear. We investigated the associations between bone turnover markers, BMD, and previous fragility fracture in a cohort of kidney transplantation candidates with advanced CKD. We hypothesized that patients with prevalent fracture would have reduced BMD and increased levels of bone turnover markers.

## Methods

### Study participants

From February 2011 through January 2014 we enrolled adult kidney transplantation candidates from four centers in Denmark. Inclusion criteria were advanced CKD and need of cardiac evaluation before kidney transplantation based on at least one of the following characteristics: age > 40 years, diabetes mellitus, maintenance dialysis therapy >5 years, kidney transplant waiting list >3 years, or symptoms of cardiovascular disease. Patients with unstable angina pectoris were excluded.

### Bone density measurements

Angiographic computed tomography (CT)-scans of chest, abdomen, and pelvis were performed on a dual-source CT-scanner (SOMATOM Definition Flash; Siemens, Erlangen, Germany). Details have been given previously [13]. Tube energy was 100–120 kVp, tube current was set at 250 mAs, and slice thickness was 3.0 mm. Intravenous x-ray contrast media was administered at a set dose of 95 mL (ioversol; Optiray, Mallinckrodt, Germany). Images were reconstructed with a standard soft tissue kernel (B30f; Syngo.via, Siemens, Erlangen, Germany).

Volumetric BMD of the lumbar spine and proximal femur was determined using *QCT Pro* version 5.1 (Mindways Software Inc., TX, US). The calibration phantom Mindways Solid (Mindways Software Inc., TX, US) was scanned at regular intervals to provide calibration data for asynchronous analysis [14]. Analysis of lumbar spine BMD was performed by placing an oval region of interest in the anterior part of three consecutive vertebrae, excluding the posterior venous plexus and avoiding focal heterogeneities and lesions. L1-L3 was preferred, but in 17 patients L2-L4 was analyzed due to visible deformities of L1. Analysis of the proximal femur was performed using the semi-automatic function provided by the software. The left hip was preferred, but in 13 patients the right hip was analyzed due to previous fracture, metallic prosthesis, or incomplete image of the left hip. *T*- and *Z*-scores were determined by reference data supplied by the software manufacturer. The two-dimensional hip projection (*CTXA*) has been recommended for use in diagnosing osteoporosis [15]. Coefficients of variations (CV) were 0.68% at the lumbar spine, 1.85% at the total hip and 2.30% at the femoral neck.

### Fracture status

Previous fractures were determined by patient interview and chart review and classified as fragility fractures if resulting from trauma equivalent to a fall from standing height, or less [16]. High-trauma fractures and fractures of fingers, toes, face, and skull were excluded. All fractures were confirmed by radiographs or radiology reports.

Prevalent VFs were diagnosed from sagittal two-dimensional reconstructions of CT images of the thoracolumbar spine (C4-L5). One reader (HSJ) reviewed all images and flagged vertebrae with ≥20% height reduction. VFs were then confirmed and classified according to Genant’s method by an experienced radiologist [17, 18].

### Biochemical measurements

Blood samples were collected in the morning after an overnight fast. Intact parathyroid hormone (PTH), alkaline phosphatase, phosphate, and ionized calcium were analyzed by standardized methods throughout the study period. Blood samples for analysis of bone turnover markers were stored at −80 °C and analyzed in a single batch upon study completion. 25-OH-Vitamin D_2_ + D_3_ was measured using tandem mass spectrometry. Bone specific alkaline phosphatase (BSAP) was measured using enzyme immunoassay (MicroVue BAP EIA Kit, Quidel®, San Diego, CA, US) with a CV of 6%; C-terminal telopeptide of type I collagen (CTX) was measured by sandwich immunometric assay with a CV of 6%; N-terminal telopeptide of type I collagen (NTX) was measured by competitive immunometric assay with a CV of 10%. P1NP trimer was measured by radioimmunoassay (IDS-iSYS, Fountain Hills, AZ, US), with a CV of 10%, and Tartrate resistant alkaline phosphatase type 5b (TRAP5b) was measured using ELISA (Quidel®, Tecomedical Group, Switzerland), and intra- and inter-assay CVs were both 3%.

### Statistical analyses

All statistical analyses were performed with standard software package STATA/IC 13.1 for Windows (StataCorp LP, TX, US). Continuous variables were visually evaluated for normal distribution by QQ-plots. Skewed variables were transformed to their natural logarithm to enable parametric statistical testing. Normally distributed variables are presented as mean ± standard deviation (SD) with 95% confidence interval (CI) and skewed variables as median with interquartile range (IQR). Differences in continuous variables were evaluated by Student’s *t* test, and dichotomous variables were tested by Pearson’s *Χ* ^*2*^ test. Differences across categorical variables were evaluated by one-way ANOVA. Multiple logistic regression analysis was utilized to adjust for potential confounders with fracture as the dichotomous outcome. Associations between biochemical markers and BMD were evaluated by Spearman’s univariate correlation followed by multiple linear regression analysis. For all analyses, a two-sided *p*-value < 0.05 was considered statistically significant.

## Results

### Characteristics of study participants

Of the 167 patients included, ten did not complete the CT-scan due to withdrawn consent (*n* = 5), cardiovascular event (*n* = 4), or kidney transplantation (*n* = 1); leaving 157 patients for the final analysis. Blood samples were missing for eight patients, and these were excluded from statistical analyses regarding bone turnover markers.

Underlying causes of CKD were: diabetes mellitus (DM) type 1 or 2 (26%), hypertension or glomerulosclerosis (25%), glomerulonephritis (24%), adult polycystic kidney disease (13%), and other/unknown (11%). Fifty-nine patients were on maintenance dialysis therapy, defined as >3 months of treatment, with a median time on dialysis of 24 months (IQR 6 to 60). The remaining 98 pre-dialysis patients had a median estimated glomerular filtration rate (eGFR) of 11 ml/min (IQR 9 to 14]. Characteristics of patients by stage of CKD are given in Additional file 1: Table S1.

One patient had recently initiated bisphosphonate treatment, and three patients were on hormone replacement therapy; none of them had a previous fragility fracture. Two patients received calcimimetics, one of which had a prevalent VF.

### Fracture

The prevalence of fragility fracture was 18%, with 55 fractures in 28 patients. Fractures included 32 VFs in 17 patients (16 grade I, 15 grade II, and 1 grade III) and 23 non-VFs in 16 patients (9 wrist, 4 ankle, 4 hip, 3 femur, 2 Charcot’s foot with osteonecrosis, and 1 clavicle fracture). Multiple fractures were seen in 12 patients, among them 5 patients with both VF and non-VF. Median time from non-VF to study inclusion was 3.9 years (range 0.2 to 16.2).

Characteristics of study participants by fracture status are shown in Table 1. Patients with prevalent fractures had reduced levels of phosphate (*p* = 0.04) and were more likely to have received prednisolone treatment (*p* = 0.03), or to be currently treated with prednisolone (*p* < 0.01). There were no differences in the levels of bone turnover markers between patients with or without prevalent fracture.

**Table 1:** Characteristics of kidney transplantation candidates by fracture status

*Characteristic*	All participants (*n* = 157)	No fracture (*n* = 129)	Previous fragility fracture (*n* = 28)	*p*
Age, years	54 [45, 63]	53 [46, 63]	57 [41, 65]	0.96
Weight, kg	77.5 (14.7)	78.2 (15.2)	74.0 (12.3)	0.18
BMI, kg/m^2^	25.7 (4.2)	25.9 (4.4)	24.8 (3.5)	0.25
Female	50 (32%)	39 (30%)	11 (39%)	0.35
Caucasian	147 (94%)	119 (92%)	28 (100%)	0.21
Active smoker	48 (31%)	40 (31%)	8 (29%)	0.80
Type 1 Diabetes	35 (22%)	25 (19%)	10 (36%)	0.08
Type 2 Diabetes	15 (10%)	14 (11%)	1 (4%)	0.48
Current dialysis therapy	59 (38%)	50 (39%)	9 (32%)	0.67
Previous kidney transplantation	28 (18%)	22 (17%)	6 (21%)	0.59
Previous prednisolone treatment	46 (29%)	33 (26%)	13 (46%)	0.03
Active prednisolone treatment	21 (13%)	12 (9%)	9 (32%)	0.004
Phosphate binder, any type	112 (71%)	90 (70%)	22 (79%)	0.49
Phosphate binder, calcium-containing	83 (53%)	65 (50%)	18 (64%)	0.21
25-OH-vitamin D supplements	39 (25%)	28 (22%)	11 (39%)	0.05
Vitamin D receptor activators	110 (70%)	90 (70%)	20 (71%)	1.00
Parathyroid hormone, ρmol/L	20.6 [13.8, 30.4]	21.3 [14.2, 31.8]	17.4 [13.7, 28.6]	0.96
Ionized calcium, mmol/L	1.22 (0.08)	1.22 (0.08)	1.21 (0.08)	0.49
Phosphate, mmol/L	1.57 (0.38)	1.60 (0.39)	1.43 (0.30)	0.04
Alkaline phosphatase, U/L	71 [56, 91]	70 [55, 91]	81 [60, 94]	0.64
25-OH-vitamin D_2_ + D_3_, nmol/L	82 (48)	83 (49)	77 (42)	0.54
Bone specific alkaline phosphatase, U/L	26 [20, 35]	26 [20, 39]	27 [22, 31]	0.77
Procollagen type 1 N-terminal propeptide, μg/L	62 [42, 91]	65 [40, 96]	60 [49, 88]	0.89
Tartrate resistant alkaline phosphatase, U/L	4.29 [2.75, 5.80]	4.12 [2.69, 5.65]	4.58 [3.70, 6.58]	0.35
C-terminal telopeptide of type I collagen, ng/mL	1.13 [0.73, 1.65]	1.15 [0.77, 1.63]	0.91 [0.68, 1.68]	0.72
N-terminal telopeptide of type I collagen, nmol/L	74 [44, 111]	75 [44, 111]	72 [49, 111]	0.99

Data are mean (SD), median [IQR], or *n* (%) and *p* = Student’s *t* test

### Bone turnover markers

Results of univariate correlation analyses between bone turnover markers and demographic variables are shown in Table 2. Several of the bone turnover markers correlated negatively with age and with body size. Both formative and resorptive markers correlated positively with PTH and alkaline phosphate levels. CTX and NTX were the only markers correlated with eGFR.

**Table 2:** Univariate correlations between biochemical markers of bone turnover and characteristics of adult kidney transplantation candidates

	BSAP		P1NP		TRAP 5b		CTX		NTX	
	*rho*		*rho*		*rho*		*rho*		*rho*	
Age, yrs	−0.16	(†)	−0.18	†	0.01		−0.19	†	−0.19	†
Weight, kg	−0.18	†	−0.22	†	−0.14	(†)	−0.14	(†)	−0.18	†
Body mass index, kg/cm^2^	−0.17	†	−0.22	†	−0.11		−0.15	(†)	−0.17	†
Estimated glomerular filtration rate (pre-dialysis only, *n* = 98)	0.16		−0.01		−0.00		−0.26	†	−0.30	†
Parathyroid hormone, ρmol/L	0.30	‡	0.37	‡	0.45	‡	0.42	‡	0.45	‡
Ionized calcium, mmol/L	−0.18	†	−0.23	†	−0.12		−0.17	†	−0.17	†
Phosphate, mmol/L	−0.11		−0.03		−0.10		0.04		0.13	
Alkaline phosphatase, U/L	0.72	‡	0.43	‡	0.35	‡	0.38	‡	0.34	‡
25-OH-vitamin D_2_ + D_3_, nmol/L	−0.02		−0.04		0.09		−0.05		−0.08	
Lumbar spine BMD, mg/cm^3^	0.05		0.07		−0.08		0.03		−0.04	
Lumbar spine *Z*-score	0.01		0.01		−0.06		−0.05		−0.13	
Total hip BMD, mg/cm^3^	−0.20	†	−0.12		−0.20	†	−0.09		−0.15	(†)
Total hip *Z*-score	−0.27	‡	−0.22	†	−0.19	†	−0.12		−0.20	†
Femoral neck BMD, mg/cm^3^	−0.10		−0.04		−0.12		0.01		−0.09	
Femoral neck *Z*-score	−0.22	†	−0.22	†	−0.18	†	−0.11		−0.20	†

Data are Spearman’s *rho* with corresponding *p*-values, (†) = *p* < 0.10, † = *p* < 0.05, ‡ = *p* < 0.001

*Abbr.*: *BMD* Bone mineral density, *BSAP* Bone specific alkaline phosphatase, *P1NP* Procollagen type 1 N-terminal propeptide, *TRAP5b* Tartrate resistant alkaline phosphatase type 5b, *CTX* C-terminal telopeptide of type I collagen, *NTX* N-terminal telopeptide of type I collagen

Patients with type 1 DM had 18% (CI 5 to 30%, *p* = 0.02) higher levels of BSAP than patients without diabetes. No other differences in levels of bone turnover markers were found between diabetics and non-diabetics (Additional file 1: Table S2).

**Fig. 1:**
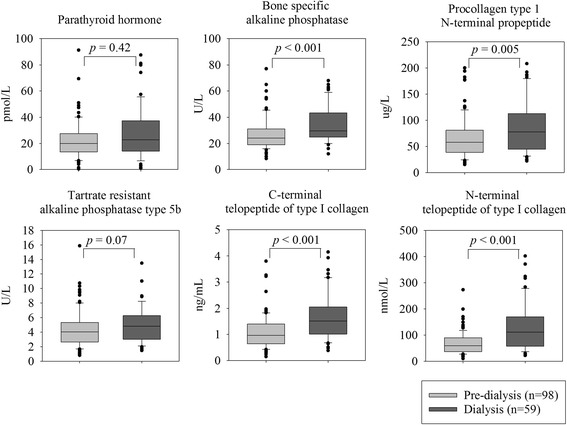
Levels of bone turnover markers in kidney transplantation candidates by dialysis status. Boxplots with median and interquartile range, whiskers at 5 and 95%, *p* = Student’s *t* test

All bone turnover markers except TRAP5b were significantly higher in patients on dialysis than in pre-dialysis patients (Fig. 1). Levels were 22% (CI 11 to 32%, *p* < 0.001) higher for BSAP; 25% higher (CI 8 to 38%, *p* = 0.005) for P1NP; 36% higher (CI 23 to 47%, *p* < 0.001) for CTX and 43% higher (CI 29 to 54%, *p* < 0.001) for NTX. Corresponding numbers for TRAP5b were 16% (CI −2 to 30%, *p* = 0.07).

### Bone density

**Fig. 2:**
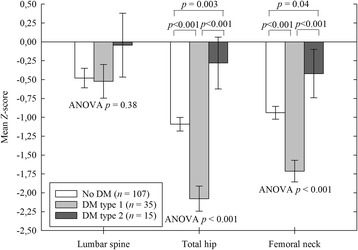
*Z*-scores of spine and hip in kidney transplantation candidates with and without diabetes mellitus. Data are mean with standard errors, ANOVA *p* = one-way analysis of variance, *p* = student’s *t* test

The number of patients with BMD below expected for their age (*Z*-score < −2.0), was 31 (20%) at the total hip, 24 (15%) at the femoral neck, and 13 (8%) at the lumbar spine. Patients with type 1 DM had lower *Z*-scores at the hip compared with non-diabetics in un-adjusted analyses, while the opposite was true for patients with type 2 DM (Fig. 2). Both DM type 1 (*β* = −0.95, *p* = <0.001) and DM type 2 (*β* = 0.72, *p* = 0.01) remained significantly associated with total hip *Z*-score in a multiple linear regression model including BMI and dialysis status. The difference between type 1 and type 2 DM was also significant in the adjusted analysis (*p* < 0.001). There were no differences in *Z*-scores between pre-dialysis and maintenance dialysis patients (Additional file 1: Table S1).

All of the markers, except CTX, correlated negatively with total hip and femoral neck *Z*-scores (Table 2). To investigate this association further, we performed multiple linear regression analyses with *Z*-score as the outcome variable, and each bone turnover marker entered individually as the main explanatory variable. As potential confounders, we included BMI, DM type 1, DM type 2, and dialysis therapy. Results are shown in Table 3. PTH was the only biochemical marker significantly associated with *Z*-scores of both spine and hip. None of the bone turnover markers were associated with lumbar spine *Z*-score, while all were negatively associated with *Z*-score at total hip. BSAP was the only marker significantly associated with total hip *Z*-score independently of PTH (*β* = −0.415, *p* = 0.04).

**Table 3:** Association between bone turnover markers and bone density in kidney transplant candidates

	Lumbar spine *Z*-score		Total hip *Z*-score		Femoral neck *Z*-score	
	*β*	*p*	*β*	*p*	*β*	*p*
Parathyroid hormone, pmol/L	−0.243	0.04	−0.271	<0.01	−0.182	0.02
Bone specific alkaline phosphatase, U/L	−0.066	0.78	−0.557	<0.01	−0.428	0.02
Procollagen type 1 N-terminal propeptide, μg/L	0.010	0.94	−0.314	0.01	−0.288	0.01
Tartrate resistant alkaline phosphatase, U/L	−0.124	0.48	−0.325	0.01	−0.285	0.02
C-terminal telopeptide of type I collagen, ng/mL	−0.107	0.52	−0.267	0.04	−0.194	0.09
N-terminal telopeptide of type I collagen, nmol/L	−0.186	0.44	−0.297	<0.01	−0.226	0.03

Multivariate linear regression coefficients, *β*, per 10% increase in biochemical marker, adjusted for body mass index, diabetes type 1, diabetes type 2, and dialysis therapy

### Bone density and fracture

**Fig. 3:**
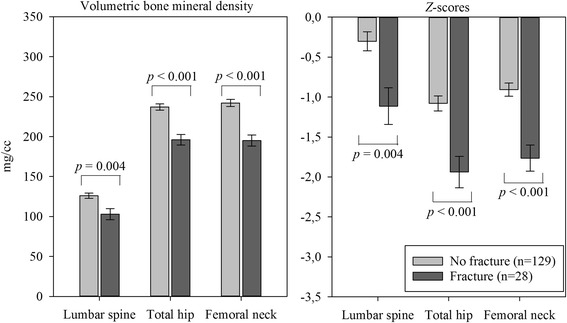
Bone density and *Z*-scores in kidney transplantation candidates with and without fragility fracture. Mean values with standard errors, *p* = Student’s t test

Measurements of bone density and bone strength are shown in Table 4. Patients with prevalent fractures had reduced bone density at all three areas in the unadjusted analysis (Fig. 3). BMD was mainly reduced at the lumbar spine in patients with VF, and at the proximal femur in patients with non-VF. Buckling ratio was increased in patients with fracture, while there was no difference in the cross-sectional moment of inertia.

**Table 4:** Bone density measurements in adult kidney transplantation candidates with and without prevalent fragility fracture

	No fracture (*n* = 129)	Any fracture (*n* = 28)		Vertebral fracture (*n* = 17)		Non-vertebral fracture (*n* = 16)	
Volumetric BMD, mg/cm^3^							
Lumbar spine	126 ± 38	103 ± 37	†	90 ± 28	‡	106 ± 44	(†)
Total hip	237 ± 43	196 ± 35	‡	201 ± 33	†	184 ± 33	‡
Femoral neck	242 ± 50	195 ± 37	‡	198 ± 37	†	180 ± 34	‡
Areal BMD, mg/cm^2^							
Total hip	0.71 ± 0.12	0.61 ± 0.10	‡	0.63 ± 0.10	†	0.56 ± 0.09	‡
Femoral neck	0.60 ± 0.10	0.51 ± 0.09	‡	0.52 ± 0.10	†	0.46 ± 0.07	‡
*Z*-score							
Lumbar spine	−0.30 ± 1.37	−1.11 ± 1.21	†	−1.29 ± 0.84	†	−0.96 ± 1.46	
Total hip	−1.08 ± 1.05	−0.94 ± 1.04	‡	−1.45 ± 0.80		−2.42 ± 0.88	‡
Femoral neck	−0.91 ± 0.94	−1.77 ± 0.86	‡	−1.41 ± 0.79		−2.26 ± 0.60	‡
*T*-score							
Lumbar spine	−1.77 ± 1.43	−2.60 ± 1.44	†	−3.13 ± 1.04	‡	−2.44 ± 1.71	
Total hip	−1.79 ± 1.05	−2.73 ± 0.89	‡	−2.47 ± 0.84	†	−3.17 ± 0.78	‡
Femoral neck	−1.74 ± 0.93	−2.60 ± 0.79	‡	−2.44 ± 0.86	†	−3.04 ± 0.64	‡
Femoral neck strength							
Buckling ratio	9.52 ± 3.05	12.9 ± 3.8	‡	12.7 ± 3.8	‡	14.1 ± 4.0	‡
Cross-sectional moment of inertia, mm^4^	7.50 ± 2.39	7.77 ± 3.33		8.52 ± 3.69		6.94 ± 3.66	

Data are mean ± SD, Student’s *t* test: † = *p* < 0.05 and ‡ = *p* < 0.001 compared to non-fractured patients

To adjust for potential confounders, we performed a multiple logistic regression analysis with prevalent fracture as the outcome variable and *Z*-score, BMI, dialysis therapy, DM type 1, and DM type 2 as explanatory variables. The odds ratio (OR) of prevalent fracture by a 1 unit decrease in *Z*-score was 1.74 (CI 1.17 to 2.60, *p* = 0.007) at the lumbar spine, 2.30 (CI 1.37 to 3.87, *p* = 0.002) at the total hip, and 3.21 (CI 1.72 to 5.98, *p* < 0.001) at the femoral neck.

## Discussion

In this cohort of kidney transplantation candidates with advanced CKD, patients with prevalent fragility fractures had reduced bone density, but they did not have increased levels of bone turnover markers. Bone turnover markers were negatively associated with bone density at the hip, but not at the spine – and this association could partly be explained by the PTH level.

### Determinants of fracture

Markers of bone turnover were not associated with fracture status. This finding is in contrast to results from the study by Nickolas et al. where both formative (osteocalcin, P1NP trimer) and resorptive (CTX, TRAP5b) markers were elevated in pre-dialysis patients with prevalent fractures [11]. There are some notable differences between these two cohorts. In the abovementioned study, patients were older, (median age of 78 and 69 years for patients with and without fracture compared with 54 years in our cohort); they had better preserved kidney function (median eGFR 25 and 28 for patients with and without fracture compared with 11 ml/min/m^3^ in our cohort), and none were on dialysis. Less than 30% received active vitamin D-compounds, compared with >70% in our cohort, and median levels of PTH were also higher among our patients. Thus, the relationship between bone turnover markers and prevalent fracture may differ depending on the severity of CKD and the extent of renal osteodystrophy. In line with this, another study including 70 patients with CKD stage 5D on hemodialysis also failed to detect a difference in the levels of BSAP and CTX based on fracture-status [19]. On the other hand, high levels of alkaline phosphatase [20] and BSAP [21] have been shown to predict fracture in Japanese hemodialysis patients. More research is needed on this topic before any firm conclusions can be drawn.

Bone density of both spine and hip was reduced in patients with prevalent fractures. This finding is in agreement with several recent observational studies, demonstrating a robust association between low BMD and an increased prevalence of fracture [22–26]. In prospective studies, low BMD increased the risk of fracture both in pre-dialysis CKD [27] and in hemodialysis patients [21]. Based on these studies, international guidelines on fracture risk assessment in CKD are currently being revised [28].

### Determinants of BMD

High levels of both formative and resorptive markers were associated with reduced BMD at the total hip, but not at the lumbar spine. These results are in agreement with previous studies. Increased levels of bone turnover markers were associated with decreased BMD [29], loss of BMD [30–32], and worsening parameters of bone quality [12] at the forearm, another distal skeletal site. Both P1NP and TRAP5b were associated with reduced BMD of hip and forearm, but not the lumbar spine in CKD-patients [11]. In a recent prospective study, TRAP5b predicted loss of BMD at the total hip in a hemodialysis cohort [33].

Further, all markers were positively correlated with levels of PTH, which also concurs with previous findings [11, 29, 30, 34, 35]. Prospectively, high levels of both PTH and bone turnover markers were associated with deterioration of cortical bone at the forearm measured by high resolution peripheral QCT [12]. In our study, the associations between BMD and bone turnover markers were attenuated when PTH-levels were taken into account.

These results support the hypothesis of secondary hyperparathyroidism as a cause of high bone turnover and subsequent loss of bone density in CKD, particularly at the peripheral skeleton [6]. Sustained hyperparathyroidism cause a high turnover state in bone, where bone resorption and, to a lesser degree, bone formation is stimulated, resulting in the release of both formative and resorptive markers [36]. The disturbed osteoblast activity leads to disorganized bone formation, with an increase in the non-mineralized component of bone [37, 38]. This could potentially be captured by BMD, which expresses mineralized bone per measured area or volume. However, few studies have attempted to relate histomorphometric parameters with BMD, and none of them recent. One study found that high turnover was related to a higher lumbar spine BMD [39], while two others reported that BMD was lower in high turnover [40, 41], particularly at the distal skeleton [41].

### Stage of CKD and bone disease

The levels of bone turnover markers were higher in patients receiving dialysis treatment than in pre-dialysis patients, despite the poor kidney function (median eGFR of 11) in our pre-dialysis patients. This inverse relationship between bone turnover markers and kidney function was also seen across CKD stages 1 to 5 [34]. CTX and NTX are cleared through the kidneys [42], and thus elevated levels are expected as CKD progresses. In contrast, the P1NP trimer, BSAP, and TRAP5b levels are believed to be unaffected by the glomerular filtration rate [43]. The elevated levels of these markers are therefore likely due to an increasing severity of bone disease after initiation of dialysis treatment. In contrast, we found no differences in BMD between pre-dialysis and maintenance dialysis patients, which could suggest that bone turnover markers are better early markers of the progression of bone disease during the transition from stage 5 to 5D of CKD.

### Diabetes

Patients with type 1 diabetes had lower and type 2 diabetics higher *Z*-scores at the hip compared to patients without diabetes, and these differences were independent of body size and stage of CKD. Insulin-dependent diabetes mellitus has been associated with reduced BMD in patients with normal kidney function [44] and also in pre-dialysis CKD patients [34]. Bone anabolic effects of insulin have been suggested as a possible mechanism behind these differences between type 1 and type 2 diabetics [45]. Another explanation could be the duration of disease, as Type 1 diabetes manifests earlier – and a debut of CKD at a younger age has been linked to reduced BMD [34]. Regardless of the underlying mechanism, we would advise that type 1 and type 2 DM be considered separately when investigating renal bone disease.

### Strengths and limitations

We consider the use of QCT for BMD analysis a strength of this study. Though DXA remains the reference standard, the QCT Pro CTXA hip projection has been accepted for use in diagnosing osteoporosis [15]. QCT may also hold certain advantages over DXA; the three-dimensional imaging enable a precise placement of the region of interest into the bone compartment, avoiding artefacts from surrounding tissues. In CKD, calcification of the abdominal aorta [46], dialytic peritoneal fluid [47], and mineral-containing phosphate binders [48] may overestimate lumbar spine aBMD by DXA. A recent study suggested that QCT was more sensitive than DXA in detecting changes in BMD over time in hemodialysis patients [33].

We did not have the opportunity to compare our QCT results with the DXA reference standard, nor did we perform bone biopsies for the diagnosis of bone turnover. Other major limitations of this study include the cross-sectional design and the lack of power to assess the relationship between fracture as a dichotomous outcome and multiple clinical factors. Our cohort showed great heterogeneity in duration, cause, and stage of CKD, which may have limited our ability to detect relevant associations. Further, as our patients were considered candidates for either living- or deceased donor kidney transplantation, they may differ substantially from an unselected CKD population at the same stage. Lastly, as the majority of our patients were Caucasian, the results may not be applicable to other ethnic groups.

## Conclusions

Our results support the hypothesis that increased bone remodeling due to hyperparathyroidism lead to reduced bone density in late stage CKD, which may contribute to an increased risk of fracture. Prospective studies are needed to further examine the utility of bone turnover markers as tools of fracture risk prediction in CKD.

## Additional file

Additional file 1: Table S1. Demographic data of kidney transplantation candidates by stage of chronic kidney disease. **Table S2.**Characteristics of kidney transplantation candidates with and without diabetes mellitus. (DOCX 22 kb)

## Abbreviations

BMD: Bone mineral density
BMI: Body mass index
BSAP: Bone specific alkaline phosphatase
CKD: Chronic kidney disease
CT: Computed tomography
CTX: C-terminal telopeptide of type 1 collagen
DM: Diabetes mellitus
eGFR: Estimated glomerular filtration rate
ELISA: Enzyme-linked immunosorbent assay
NTX: N-terminal telopeptide of type 1 collagen
P1NP: Procollagen type 1 N-terminal pro-peptide
PTH: Parathyroid hormone
TRAP5b: Tartrate resistant alkaline phosphatase type 5b
VF: Vertebral fracture
