# Supplementary material for: Bone turnover markers are associated with bone density, but not with fracture in end stage kidney disease: a cross-sectional study
Source: BMC Nephrol. 2017 Sep 6;18:284. doi: 10.1186/s12882-017-0692-5 (PMC5586067; doi:10.1186/s12882-017-0692-5)
Supplement: Additional file 1: Table S1. — Demographic data of kidney transplantation candidates by stage of chronic kidney disease. Table S2.Characteristics of kidney transplantation candidates with and without diabetes mellitus. (DOCX 22 kb) [file 12882_2017_692_MOESM1_ESM.docx]

## Supplementary Tables

| Supplementary Table 1 Demographic data of kidney transplant candidates by stage of chronic kidney disease | | | |
| --- | --- | --- | --- |
| *Characteristic* | Pre-dialysis  (*n* = 98) | Maintenance dialysis  (*n* = 59) | *p* |
| Age, years | 54 (11) | 54 (12) | 0.92 |
| Weight, kg | 78 (15) | 77 (15) | 0.93 |
| BMI, kg/m² | 25.7 (4.0) | 25.7 (4.7) | 0.95 |
| Female | 30 (31%) | 20 (34%) | 0.73 |
| Type 1 Diabetes Mellitus | 28 (29%) | 7 (12%) | 0.01 |
| Type 2 Diabetes Mellitus | 9 (9%) | 6 (10%) | 1.00 |
|  |  |  |  |
| Phosphate binder, any type | 59 (60%) | 53 (90%) | <0.001 |
| Phosphate binder, calcium-containing | 45 (46%) | 38 (64%) | 0.03 |
| 25-OH-vitamin D supplements | 27 (28%) | 12 (20%) | 0.31 |
| Vitamin D receptor activators | 67 (68%) | 43 (73%) | 0.55 |
|  |  |  |  |
| Parathyroid hormone, ρmol/L | 20.0 [13.6, 27.5] | 22.7 [14.1, 37.3] | 0.42 |
| Ionized calcium, mmol/L | 1.21 (0.08) | 1.23 (0.09) | 0.07 |
| Phosphate, mmol/L | 1.55 (0.35) | 1.59 (0.43) | 0.57 |
| Alkaline phosphatase, U/L | 67 [52, 88] | 78 [61, 99] | 0.03 |
| 25-OH-vitamin D₂+D₃, nmol/L | 81.41 (46.7) | 82.78 (50.0) | 0.87 |
|  |  |  |  |
| Bone specific alkaline phosphatase, U/L | 24 [19, 31] | 30 [25, 43] | <0.001 |
| Procollagen type 1 N-terminal propeptide, μg/L | 57.9  [38.8, 81.0] | 78.0  [45.0, 112.8] | 0.005 |
| Tartrate resistant alkaline phosphatase, U/L | 4.05  [2.66, 5.33] | 4.81  [3.20 6.28] | 0.053 |
| C-terminal telopeptide of type I collagen, ng/mL | 0.97  [0.64, 1.40] | 1.52  [1.01, 2.05] | <0.001 |
| N-terminal telopeptide of type I collagen, nmol/L | 59 [37, 90] | 112 [58, 170] | <0.001 |
|  |  |  |  |
| Lumbar spine *Z*-score | -0.50 (1.16) | -0.35 (1.65) | 0.51 |
| Total hip *Z*-score | -1.29 (1.11) | -1.15 (1.08) | 0.45 |
| Femoral neck *Z*-score | -1.09 (0.96) | -1.01 (1.02) | 0.62 |
| Mean (SD), median [IQR] or *n* (%), *p* = Student's *t* test or Pearson’s *Χ*² test | | | |

| Supplementary Table 2 Characteristics of kidney transplant candidates with and without diabetes mellitus | | | | | |
| --- | --- | --- | --- | --- | --- |
| *Characteristic* | No diabetes  (*n* = 107) | Type 1 diabetes (*n* = 35) | Type 2 diabetes (*n* = 15) | *p* |  |
| Age, yrs | 57 (10.3) | 46 (7.5)† ‡ | 55 (12.5) | <0.001 |  |
| Weight, kg | 78 (14) | 72 (15) †‡ | 89 (10) † | <0.001 |  |
| Body mass index, kg/m² | 25.8 (3.9) | 23.9 (4.2) †‡ | 29.1 (4.6) † | <0.001 |  |
| Female | 37 (35%) | 11 (31%) | 2 (13%) | 0.25 |  |
| Previous kidney transplantation | 22 (21%) | 5 (14%) | 1 (7%) | 0.35 |  |
| Previous prednisolone treatment | 36 (34%) | 4 (11%) | 2 (13%) | 0.02 |  |
| Phosphate binder, any type | 74 (69%) | 27 (77%) | 11 (73%) | 0.70 |  |
| 25-OH-vitamin D supplements | 24 (22%) | 9 (26%) | 6 (40%) | 0.33 |  |
| Vitamin D receptor activators | 76 (71%) | 21 (60%) | 13 (87%) | 0.16 |  |
|  |  |  |  |  |  |
| Parathyroid hormone, ρmol/L | 20.6  [13.3, 23.3] | 18.1  [13.2, 19.9] | 23.3  [18.3, 25.3] | 0.39 |  |
| Ionized Calcium, mmol/L | 1.23 (0.07) | 1.20 (0.09) | 1.21 (0.12) | 0.38 |  |
| Phosphate, mmol/L | 1.54 (0.38) | 1.64 (0.38) | 1.59 (0.33) | 0.36 |  |
| Alkaline Phosphatase, U/L | 67 [51, 71] | 84 [64, 89] † | 73 [56, 78] | 0.02 |  |
| 25-OH-Vitamin D₂+D₃, nmol/L | 87.9 (47.1) | 67.5 (49.5) | 72.4 (42.6) | 0.07 |  |
|  |  |  |  |  |  |
| Lumbar spine *Z*-score | -0.53 (1.22) | -0.52 (1.32) | -0.04 (1.64) | 0.38 |  |
| Total hip *Z*-score | -1.09 (0.92) | -2.08 (0.98) †‡ | -0.28 (1.33) † | <0.001 |  |
| Femoral neck *Z*-score | -0.94 (0.87) | -1.71 (0.85) †‡ | -0.42 (1.24) † | <0.001 |  |
|  |  |  |  |  |  |
| Bone specific alkaline phosphatase, U/L | 24 [20, 32] | 30 [24, 45] † | 29 [25, 34] | 0.03 |  |
| Procollagen type 1 N-terminal propeptide, μg/L | 61.0  [39.9, 85.3] | 77.8  [45.5, 101.6] | 72.5  [50.5, 106.8] | 0.59 |  |
| Tartrate resistant alkaline phosphatase, U/L | 3.97  [2.60, 5.73] | 6.19  [4.40, 0.65] | 4.88  [4.07, 6.16] | 0.27 |  |
| C-terminal telopeptide of type I collagen, ng/mL | 1.12  [0.73, 1.65] | 1.02  [0.65, 1.63] | 1.37  [0.87, 1.83] | 0.38 |  |
| N-terminal telopeptide of type I collagen, nmol/L | 71  [43, 112] | 79.5  [38.8, 109.3] | 74.5  [54.3, 111.5] | 0.87 |  |
| Data are mean (SD), median [IQR], or *n* (%) and *p* = One-way analysis of variance with † = *p* < 0.05 compared non-diabetics, ‡ = *p* < 0.05 between type 1 and type 2 diabetics | | | | | |
